# Protective Effect of Alpha-Linolenic Acid on Human Oral Squamous Cell Carcinoma Metastasis and Apoptotic Cell Death

**DOI:** 10.3390/nu15234992

**Published:** 2023-12-01

**Authors:** Ching-Chyuan Su, Cheng-Chia Yu, Yi-Wen Shih, Kai-Li Liu, Haw-Wen Chen, Chih-Chung Wu, Ya-Chen Yang, En-Ling Yeh, Chien-Chun Li

**Affiliations:** 1 Antai Medical Care Corporation Antai Tian-Sheng Memorial Hospital, Pingtung 92842, Taiwan; a085085@mail.tsmh.org.tw; 2 Department of Beauty Science, Meiho University, Pingtung 91202, Taiwan; 3 School of Dentistry, Chung Shan Medical University, Taichung 40201, Taiwan; ccyu@csmu.edu.tw; 4 Department of Dentistry, Chung Shan Medical University Hospital, Taichung 40201, Taiwan; 5 Institute of Oral Sciences, Chung Shan Medical University, Taichung 40201, Taiwan; 6 Department of Nutrition, Chung Shan Medical University, Taichung 40201, Taiwan; 7 Department of Nutrition, Chung Shan Medical University Hospital, Taichung 40201, Taiwan; 8 Department of Nutrition, China Medical University, Taichung 40678, Taiwan; 9 Department of Food and Nutrition, Providence University, Taichung 43301, Taiwan; 10 Department of Food Nutrition and Health Biotechnology, Asia University, Taichung 41354, Taiwan; 11 Department of Nutrition, College of Medical and Health Care, Hung-Kuang University, Taichung 43302, Taiwan

**Keywords:** α-linolenic acid, epithelial–mesenchymal transition, metastasis, apoptosis, oral cancer, OSCC

## Abstract

Oral cancer ranks sixth among Taiwan’s top 10 cancers and most patients with poor prognosis acquire metastases. The essential fatty acid alpha-linolenic acid (ALA) has been found to diminish many cancer properties. However, the anti-cancer activity of ALA in oral cancer has yet to be determined. We examined the mechanisms underlying ALA inhibition of metastasis and induction of apoptotic cell death in oral squamous cell carcinoma (OSCC). Migration and invasion assays confirmed the cancer cells’ EMT capabilities, whereas flow cytometry and Western blotting identified molecular pathways in OSCC. ALA dramatically reduced cell growth in a concentration-dependent manner according to the findings. Low concentrations of ALA (100 or 200 μM) inhibit colony formation, the expression of Twist and EMT-related proteins, the expression of MMP2/-9 proteins, and enzyme activity, as well as cell migration and invasion. Treatment with high concentrations of ALA (200 or 400 μM) greatly increases JNK phosphorylation and c-jun nuclear accumulation and then upregulates the FasL/caspase8/caspase3 and Bid/cytochrome c/caspase9/caspase3 pathways, leading to cell death. Low concentrations of ALA inhibit SAS and GNM cell migration and invasion by suppressing Twist and downregulating EMT-related proteins or by decreasing the protein expression and enzyme activity of MMP-2/-9, whereas high concentrations of ALA promote apoptosis by activating the JNK/FasL/caspase 8/caspase 3-extrinsic pathway and the Bid/cytochrome c/caspase 9 pathway. ALA demonstrates potential as a treatment for OSCC patients.

## 1. Introduction

Oral cancer comprises cancers of the lips, tongue, and other oral structures, and it is the most prevalent form of head and neck cancer. According to the most recent report on cancer statistics worldwide, published in 2020 by the WHO’s International Agency for Research on Cancer (IARC) [1], oral cancer ranks 16th among malignant tumors worldwide, with a global incidence rate of 2%, approximately 378,000 people; the mortality rate is 1.8%, around 178,000 people. Both the incidence and mortality rates are highest in the Asian region. Cancer originates from genetic mutations and is mainly associated with interactions between environmental, genetic, and metabolic factors [2,3,4,5,6].

The risk factors for oral cancer include heavy tobacco use [7] and excessive alcohol consumption [8,9,10]. Smoking and alcohol consumption have been proven to be the primary risk factors for oral squamous cell carcinoma (OSCC), with a synergistic effect that increases the risk by 35 times [11]. Chewing betel nut, which contains betel nut extracts like ripe areca nut extracts (rANE) and tender areca nut extracts (tANE), increases the risk of oral cancer because it promotes the generation of reactive oxygen species (ROS) and the upregulation of the expression of inflammatory factors including cyclooxygenase-2 (COX-2), prostaglandin E2 (PGE2), and interleukin-1 (IL-1) [12,13].

Neck lymph node metastasis and the extracapsular spread (ECS) of lymph nodes are both major factors contributing to poor prognosis in oral cancer [14,15]. ECS can increase distant metastasis and mortality rates. Patients with neck metastases who are without ECS had a 52% chance of surviving for 5 years, while those with ECS have a 28% chance [16,17]. ECS tumor EMT levels can predict the prognosis and survival of head and neck cancer, with shorter overall and disease-free survival, a greater recurrence rate, and wider spread shown in tumors with a higher EMT proportion [18].

The initial phase in metastasis is the epithelial–mesenchymal transition (EMT), which can reduce cell–cell adhesion and increase tumor cell adherence to the matrix. To further their invasion and intravasation, tumor cells release matrix metalloproteinases (MMPs) to destroy the extracellular matrix (ECM). Essential steps in the initiation of EMT include the binding of these proteins to the promoter regions of cell adhesion-related genes and the subsequent regulation of the expression of EMT-related proteins [19,20]. Twist1 can activate EMT by upregulating Vimentin [19]. Twist is mainly activated via the TGF-β, Wnt/β-catenin, STAT3, and hypoxia signaling pathways, initiating EMT [21]. Studies have shown that Twist can downregulate E-cadherin and promote the expression of N-cadherin, fibronectin, and Vimentin [22]. Relevant studies have shown that the transcription factors Snail and ZEB2, associated with EMT, can upregulate MMP expression, further promoting cancer cell invasion [20]. 

Cell apoptosis is an irreversible process and is considered a unique and important programmed cell death mode that maintains cellular homeostasis within tissues. The pathways involved can be broadly classified as either extrinsic (involving death receptors) or intrinsic (involving mitochondria). Transmembrane death receptors, mostly derived from the tumor necrosis factor receptor gene superfamily, mediate the extrinsic pathway [23]. The death receptors of the TNF receptor family contain a death domain rich in cysteine residues in their extracellular structure, which binds to procaspase-8 and self-catalyzes into cleaved caspase-8. This further cleaves and activates caspase-3 and promotes the cleavage of Poly(ADP-ribose) Polymerases (PARP), leading to cell apoptosis [23]. Multiple non-receptor stimuli, such as growth factors, hormones, radiation, toxins, hypoxia, viral infection, and free radicals, are involved in the intrinsic pathway. These conditions trigger the release of cytochrome c into the cytoplasm by increasing the permeability of the mitochondrial membrane, leading to a loss of the mitochondrial membrane potential. Apoptosomes are formed when cytochrome c binds to Apaf-1 and procaspase-9 [24]. After being cleaved, procaspase-9 then activates caspase-3, which in turn induces cell death. Cytoplasmic adapter proteins with death domains are recruited by ligands and death receptors such as FasL/FasR, TNF-/TNFR1, Apo3L/DR3, Apo2L/DR4, and Apo2L/DR5. Through death domain dimerization, the Fas ligand (FasL) binds to procaspase-8 and establishes a connection with the Fas-associated death domain (FADD) [23]. As a result, procaspase-8 is cleaved and activated by the death-inducing signaling complex (DISC), which in turn activates caspase-3 and promotes cell apoptosis [23]. 

Alpha-linolenic acid (ALA, C18:3n-3), a carboxylic acid with 18 carbons and three double bonds, is an essential fatty acid needed for human health [25]. ALA comes from plant-based sources, including walnuts, flaxseeds, and hemp seeds [26]. ALA is a substrate for the synthesis of the long-chain polyunsaturated fatty acid (PUFA) eicosapentaenoic acid (EPA, C20:5n-3) and docosahexaenoic acid (DHA, C22:6n-3) [27]. ALA and its metabolites have been reported to have antioxidative, anti-inflammatory, cardiovascular-protective, neuro-protective, anti-osteoporotic, and anti-cancer effects [28]. ALA has been shown to decrease cell viability and proliferation in various types of cancer, including breast, colon, cervical, and prostate cancer cells [29,30,31,32]. However, there is limited research on its relevance to oral cancer and further investigation is warranted.

## 2. Materials and Methods

### 2.1. Cell Culture

SAS and GNM human oral squamous cell carcinoma cell lines were provided by Professor Chen-Chia Yu of Chung Shan Medical University for this study. The cells were grown in DMEM with 10% FBS and 1% penicillin added. The cells were incubated in a humidified incubator at 37 °C. Additionally, the medium was changed every one to two days.

### 2.2. Cell Viability Analysis

Cell metabolic activity was measured using the MTT assay. SAS (2.5 × 10^5^) and GNM (4 × 10^5^) cell lines were treated with serial concentrations of ALA (0–800 μM) for 24 h. Then, the cells were assessed for viability using the MTT reagent. To ascertain the results, the absorbance was detected at 570 nm using a microplate reader.

### 2.3. Total Protein Extraction

SAS (6 × 10^5^ in 6 cm dish) and GNM (1 × 10^6^ in 6 cm dish) cell lines were treated with different concentrations of ALA (0–200 μM) for different time durations. After treatment, the cells were scraped and lysed using an ultrasonic cell disruptor (Vibra-Cell™, Sonics & Materials, Inc., Newtown, CT, USA). After cell lysis, the samples were centrifuged at 4 °C and 15,000 rpm (20,600× *g*) for 30 min using a refrigerated microcentrifuge (3500× *g* Centrifuge, KUBOTA, Osaka, Japan). The resulting supernatant can be used for protein quantification, sample preparation, or stored at −20 °C.

### 2.4. Nuclear and Cytosolic Protein Extraction

SAS and GNM cell lines were seeded at densities of 3 × 10^6^ and 3.6 × 10^6^ cells in 10 cm culture dishes; treated with 200 and 400 μM of ALA for 0, 15, 30, and 60 min; and then centrifuged at 4 °C and 8500 rpm (6600× *g*) for 5 min using a refrigerated microcentrifuge. Then, the sample was subjected to 30 min of shaking at 4 °C using an orbital shaker (Intelli-Mixer RM-2, Daigger Scientific, Hamilton, NJ, USA). The cytosolic protein was extracted from the supernatant, and the nuclear protein was extracted from the pellet. Both protein extracts could be used for protein quantification, sample preparation, or stored at −20 °C.

### 2.5. Mitochondrial Protein Extraction

SAS and GNM cells were seeded at a density of 1.8 × 10^6^ and 2.1 × 10^6^, respectively, in 10 cm culture dishes and treated with various concentrations of ALA (0, 50, 100, and 200 μM) for 16 h. The cells were then harvested and cytosol was extracted using a buffer mix. The supernatant was collected and saved. The supernatant was further centrifuged at 4 °C and 10,400 rpm (10,000× *g*) for 30 min and the resulting supernatant was collected as the cytoplasmic fraction. Next, 80 μL of mitochondria extraction buffer mix was added to the Eppendorf tube containing the pellet. The tube was vortexed (Vortex-Genie 2, Scientific Industries, Inc., New York, NY, USA) for 10 s to separate the mitochondria, which could be directly used for sample preparation or stored at −20 °C in a freezer.

### 2.6. Western Blotting

A semi-dry transfer to a polyvinylidene fluoride (PVDF) membrane was used to transfer the proteins that had been separated via 10% sodium dodecyl sulfate–polyacrylamide gel electrophoresis (SDS-PAGE). The transferred PVDF membranes were blocked with 5% non-fat dry milk in PBS. Primary antibodies were selected based on the research objectives and incubated with the membranes overnight at 4 °C. The membranes were then washed three times with PBST at room temperature. Secondary antibodies were selected based on the research objectives and incubated with the membranes for 1 h at room temperature. After three washes with PBST, the antibodies bound to the protein blots were detected using ImageQuant (LAS-4000, FUJIFILM, Tokyo, Japan). The following primary antibodies and reagents were used in this study: caspase-8 antibody (9746) (1:500), Anti-phospho-JNK (Thr183/Tyr185, Thr221/Tyr223) antibody (07-175) (1:1000), β-actin antibody (MAB1501) (1:4000), Fas antibody (GTX13550) (1:1000), Fas ligand antibody (GTX66619) (1:1000), Bid antibody (GTX110568) (1:1000), E-cadherin antibody (GTX100443) (1:1000), Vimentin antibody (GTX100619) (1:5000), Twist 1/2 antibody (GTX127310) (1:1000), MMP-2 antibody (GTX104577) (1:1000), c-jun antibody (SC-1694) (1:1000), GAPDH antibody (SC-32233) (1:500), Anti-JNK 1/2 antibody (A1HO1362) (1:500), Anti-caspase 9 antibody (ab32539) (1:500), Anti-cleaved-caspase 3 antibody (ab2302) (1:500), Anti-cytochrome c antibody (ab13575) (1:1000), Anti-Poly-(ADP-Ribose)-Polymerase antibody (11835238001) (1:1000), COXIV Polyclonal antibody (11242-1-AP) (1:1000), and MMP-9 antibody (NBP2-13173) (1:1000). The following secondary antibodies were used: Mouse IgG antibody (GTX213111-01) (1:6000) and Rabbit IgG H&L antibody (ab97051) (1:6000).

### 2.7. Wound Healing Assay

Culture inserts were attached to 3 cm culture dishes and 70 μL of SAS (1.5 × 10^4^) and GNM (3 × 10^4^) cells were seeded into the grids on both sides of the culture inserts. After incubation for 24 h, 2 mL of the DMEM medium was added to each plate and the culture insert was removed vertically. The images were taken at a 100× magnification level using a microscope. Subsequently, serial concentrations of 0–100 μM or 0–200 μM of ALA were added to the dishes and they were incubated in a cell culture incubator for 24 or 48 h. The cells were observed and photographed under a microscope at 100× magnification and the images were quantified using Image J 1.54f.

### 2.8. Cell Invasion Assay

An 8.0 μm pore invasion chamber coated with Matrigel was set up and then the SAS (1.5 × 10^4^) and GNM (3 × 10^4^) cells were seeded into it. The cells were incubated for 24 h and then treated with serial concentrations of ALA (0–100 μM or 0–200 μM). After a further day of incubation, the cells were fixed and stained for microscopic examination. Image J was then used to perform the analysis of the images.

### 2.9. Analysis of Cell Apoptosis

The SAS and GNM cell lines were separately seeded in 6 cm culture dishes at a density of 6 × 10^5^ and 1 × 10^6^ cells, respectively. Serial concentrations of ALA (0–200 μM or 0–400 μM) were added to the dishes and the cells were treated for 24 h or 0, 12, or 24 h with 200 or 400 μM of ALA. The morphology of cells was examined and photographed at 100× magnification. After treatment, the cells were detached, collected, and detected via flow cytometry. Flow cytometry was used for the detection and quantification of cell apoptosis. The scatter plot displayed annexin V fluorescence-labeled cells on the *x*-axis and propidium iodide fluorescence-labeled cells on the *y*-axis. By combining annexin V and propidium iodide staining, the results could be distinguished using flow cytometry. Finally, the data were quantified using Flow JO v10.10 analysis software.

### 2.10. Cell Colony Formation Assay

The SAS and GNM cell lines were separately seeded in a 6-well plate at a density of 2 × 10^2^ and 2 × 10^3^ cells, respectively. Serial concentrations of ALA (0–100 μM or 0–200 μM) were added to the wells and then the cells were cultured for 9 days. The absorbance was measured at 645 nm using a microplate reader. It is reasonable to calculate the colony-forming fraction by comparing the absorbance readings of the control and treatment groups. After treatment, colonies were quantified using Image J software. The percentage of colonies was determined by comparing the absorbance values of the control group and the treatment group.

### 2.11. Gelatinase Zymography Analysis

The SAS and GNM cell lines were separately seeded in a 6 cm culture dish at a density of 6 × 10^5^ and 1 × 10^6^ cells, respectively. Then, the cells were treated with serial concentrations of ALA (0–200 μM or 0–400 μM) and incubated for 24 h. Electrophoresis was performed on the collected conditioned medium after it had been combined with a loading buffer. The gel was washed twice, incubated in the reaction buffer (containing 1% NaN_3_, 2 M Tris base pH 8.0, and 1 M CaCl_2_), stained (containing Coomassie Brilliant Blue R-250, naphthol blue black methanol, and acetic acid), and destained (containing methanol and acetic acid). The enzyme activity was quantified using AlphaEaseFC 4.0 Software.

### 2.12. Statistical Analysis

The experimental data for each group were presented as mean ± SD. SPSS was used for statistical analysis of the experimental data. One-way ANOVA followed by Tukey’s test was used for analysis. Significant differences between groups were denoted by different letters when the *p*-value was <0.05. The statistical data in all figures were analyzed using the *t*-test (Student’s two-tailed *t*-test) and significance was indicated by asterisks (* *p* < 0.05, ** *p* < 0.01, and *** *p* < 0.001), compared with the control. The graphs were generated using GraphPad Prism 5.0 software and the pathway diagram for the conclusion was created using BioRender (http://biorender.com).

## 3. Results

### 3.1. Effects of ALA on Cell Morphology, Cell Viability, and Cell Colony Formation in SAS and GNM Cells

**Figure 1:**
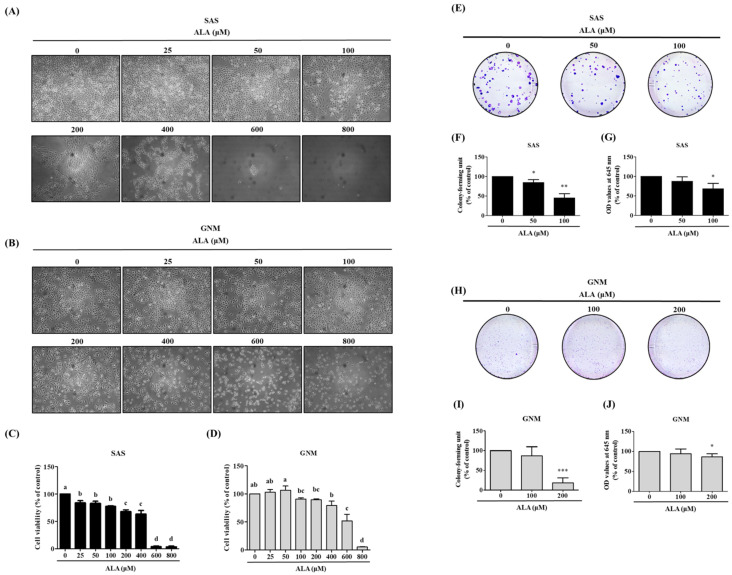
Effect of ALA on morphology and cell viability in SAS cells and GNM cells. (**A**) The morphology of SAS cells. (**B**) The morphology of GNM cells. (**C**) The cell viability was determined using the MTT assay of SAS. (**D**) The cell viability was determined using the MTT assay of GMN. (**E**–**G**) The SAS cells’ growth and proliferation were determined using the colony formation assay. (**H**–**J**) The GNM cells’ growth and proliferation were determined using the colony formation assay. Values are expressed as mean ± standard deviation. The significance of the difference in weeks of different tests was evaluated via Tukey’s multiple-range test statistical analysis. Significant differences between groups were denoted by different letters: a, b, c, and d, indicate that the results are statistically different from each other (*p* < 0.05). The difference between the control and the ALA-treated group means was compared using Student’s *t*-test (* *p* < 0.05, ** *p* < 0.01, *** *p* < 0.001).

In this study, the impact of different doses of ALA on the cell morphology and viability of SAS and GNM was observed using microscopy and an MTT assay. The results show (Figure 1A,B) that treatment with 0, 25, 50, 100, 200, 400, 600, and 800 μM of ALA for 24 h caused noticeable shrinkage and rounding of SAS and GNM cells, as well as a relative decrease in cell number, particularly at doses above 200 μM of ALA. ALA significantly reduced the cell viability of SAS (Figure 1C), with a survival rate of 67.9% at a dose of 200 μM of ALA, while at 600 μM of ALA, the survival rate was only about 3.7%. Similarly, ALA treatment significantly decreased the cell viability of GNM (Figure 1D), with a survival rate of 79.1% at a dose of 400 μM of ALA, and a survival rate of approximately 5% at 800 μM of ALA, accompanied by cell shrinkage and even suspension. Therefore, for further research, the SAS and GNM cells were treated with 0, 50, 100, and 200 μM of ALA, and 0, 100, 200, and 400 μM of ALA, respectively. After 9 days of treatment, changes in cell growth and proliferation were observed. The colony formation assay results (Figure 1E–J) revealed that as the dose of ALA increased, the number of cell colonies decreased, with the highest dose of ALA exhibiting the most significant inhibitory effect. These results indicate that ALA significantly inhibits cell proliferation and colony formation of both SAS (Figure 1E–G) and GNM (Figure 1H–J) cells.

### 3.2. Effects of ALA on Cell Migration, Invasion, and Related Protein Expression in SAS and GNM Cells

**Figure 2:**
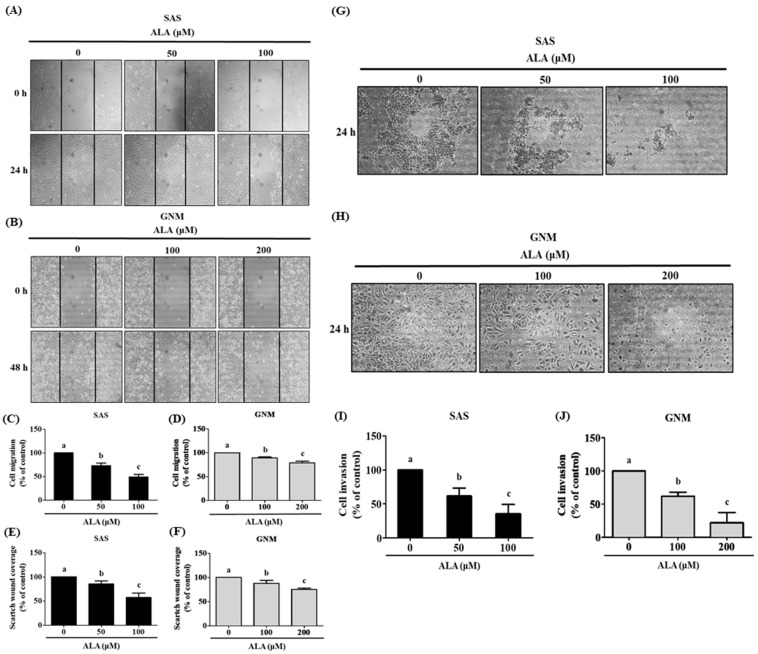
Depicts the effect of ALA on cell migration and invasion in SAS and GNM cells. SAS cells (**A**) and GNM cells (**B**) were treated for 24 or 48 h with 0, 50, and 100, or 0, 100, and 200 μM of ALA. (**C**–**F**) The wound healing assay was used to measure cell migration. (**G**) SAS cells and (**H**) GNM cells were treated for 24 h with 0, 50, and 100, or 0, 100, and 200 μM of ALA. The cell invasion was measured using the Boyden chamber assay (**I**,**J**). The mean and standard deviation are used to express the values. Tukey’s multiple range test statistical analysis was used to assess the significance of differences in weeks across different tests. Significant differences between groups were denoted by different letters: a, b, and c, indicate that the results are statistically different (*p* < 0.05).

The results of the wound-healing assay and the Boyden chamber assay were analyzed to determine if ALA affected cell migration and invasion. The results show that ALA significantly inhibited the migration of SAS and GNM cells (Figure 2A–F). Compared with the control group (100%), treatment with 50 and 100 μM of ALA reduced cell migration to 72.9% and 49.3% for SAS, respectively (Figure 2C,E), and 100 and 200 μM of ALA reduced GNM cell migration to 89.1% and 78.6%, respectively (Figure 2D,F). The Boyden chamber assay results (Figure 2G,H) reveal that ALA effectively suppressed the invasion ability of SAS and GNM cells. Compared with the control group (100%), treatment with 50 and 100 μM of ALA reduced cell invasion to 61.7% and 35.6%, respectively, while 100 and 200 μM of ALA reduced GNM cell invasion to 62.1% and 22.1%, respectively (Figure 2I,J). These results demonstrate that ALA can effectively inhibit the migration and invasion abilities of SAS and GNM cells.

**Figure 3:**
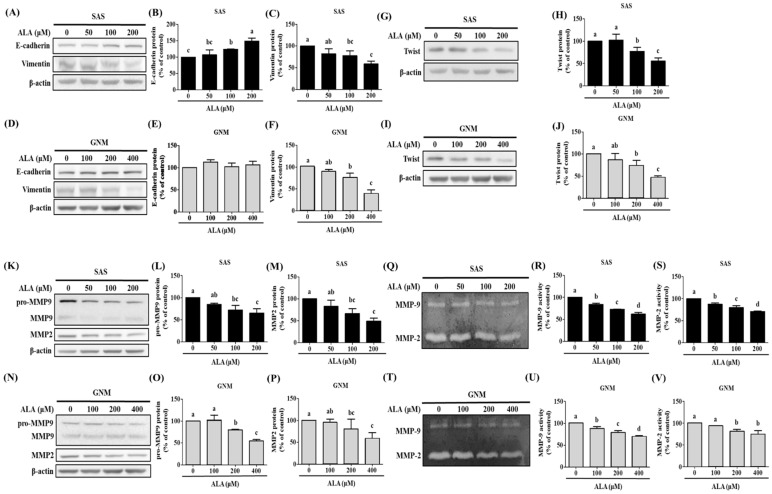
The effect of ALA on the expression of EMT-related proteins in SAS and GNM cells. For 24 h, cells were treated with 0, 50, 100, and 200 μM or 0, 100, 200, and 400 μM of ALA. (**A**) Expression of E-cadherin and vimentin in SAS cells. (**B**) SAS cell E-cadherin expression quantification. (**C**) SAS vimentin expression quantification. (**D**) Expression of E-cadherin and vimentin in GNM cells. (**E**) Quantification of E-cadherin expression in GNM cells. (**F**) Quantification of vimentin expression in GNM cells. (**G**) Twist expression in SAS cells. (**H**) SAS cell twist expression quantification. (**I**) Twist expression in GNM cells. (**J**) Quantification of twist expression in GNM cells. (**K**) SAS cell MMP-2 and MMP-9 expression. (**L**) SAS cell pro-MMP9 expression quantification. (**M**) SAS cell MMP-2 expression measurement. (**N**) Expression of MMP-2 and MMP-9 in GNM cells. (**O**) Quantification of pro-MMP9 expression in GNM cells. (**P**) Quantification of MMP-2 expression in GNM cells. (**Q**) Protein expression and enzyme activity of MMP-2 and MMP-9 in SAS cells. (**R**) MMP-2 enzyme activity in SAS cells. (**S**) MMP-9 enzyme activity in SAS cells. (**T**) Protein expression and enzyme activity of MMP-2 and MMP-9 in GNM cells. (**U**) MMP-2 enzyme activity in GNM cells. (**V**) MMP-9 enzyme activity in GNM cells. Western blotting was used to evaluate the protein expression of various proteins. Gelatin zymography was used to assess enzyme activity. The mean and standard deviation are used to express the values. Tukey‘s multiple range test statistical analysis was used to assess the significance of differences in weeks across different tests. Significant differences between groups were denoted by different letters: a, b, c and d, indicate that the results are statistically different (*p* < 0.05).

Poor tumor differentiation and high invasiveness have both been linked to the expression of EMT-related proteins in individuals with oral cancer, according to previous research [33]. Protein expression for E-cadherin and Vimentin was measured after treating SAS and GNM cells with 0, 50, 100, and 200 μM of ALA and 0, 100, 200, and 400 μM of ALA, respectively. The results show that E-cadherin expression and Vimentin expression were reduced in SAS cells when the ALA concentration increased (Figure 3A–C). Although ALA had no effect on E-cadherin expression in GNM cells, it had an inhibitory effect on Vimentin’s protein expression (Figure 3D–F). As a result, EMT in SAS and GNM cells can be successfully blocked by ALA. The expression of the protein Twist has been shown to substantially correlate with clinical stage and lymph node metastatic aggressiveness in esophageal squamous cell carcinoma [34]. The cells were treated with 0, 50, 100, and 200 μM of ALA and 0, 100, 200, and 400 μM of ALA in SAS and GNM cells, respectively (Figure 3G–J), and the expression of the Twist protein was monitored. Figure 3G–J reveal that the SAS and GNM cell Twist protein expression drastically decreased as the ALA dose increased. It has been hypothesized that ALA can suppress EMT in SAS and GNM cells by influencing Twist transcriptionally. Previous studies revealed the elevated expression and enzymatic activity of MMP-2 and MMP-9 in response to oral cancer cell motility and invasion [35]. The cells were treated with 0, 50, 100, and 200 μM of ALA and 0, 100, 200, and 400 μM of ALA, respectively, to determine if ALA influences the expression and enzymatic activity of MMP-2 and MMP-9 proteins. The results demonstrate that the expression of MMP-2 and pro-MMP-9 proteins, as well as their enzymatic activity, considerably decreases as the dose of ALA increases (Figure 3K–V). ALA may prevent SAS and GNM cell migration and invasion by decreasing MMP-2 and MMP-9 expression and activity.

### 3.3. Effects of ALA on Cell Apoptosis and Related Protein Expression in SAS and GNM Cells

**Figure 4:**
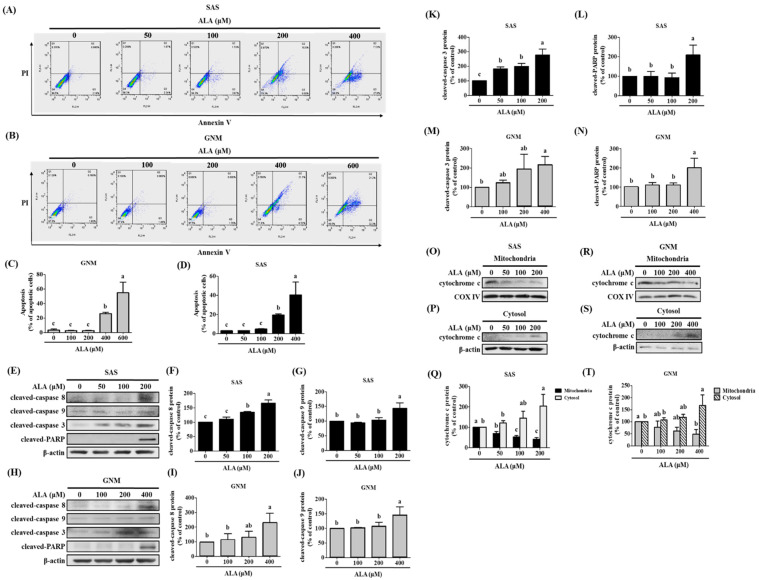
Effect of ALA on the degree of apoptosis in SAS cells and GNM cells. SAS and GNM cells were treated for 24 h with 0, 50, 100, 200, 400, or 600 μM of ALA. (**A**) The level of apoptosis in SAS cells. (**B**) Apoptosis levels in GNM cells. (**C**) SAS cell apoptosis %. (**D**) GNM cell apoptosis %. (**E**) Apoptosis-related protein expression in SAS cells. (**F**) SAS cells’ cleaved caspase 8 expression was quantified. (**G**) SAS cells’ cleaved caspase 9 expression was quantified. (**H**) Apoptosis-related protein expression in GNM cells. (**I**) Quantification of cleaved caspase 8 expression in GNM cells. (**J**) Quantification of cleaved caspase 9 expression in GNM cells. (**K**) SAS cells’ cleaved caspase 3 expression was quantified. (**L**) SAS cells’ cleaved caspase PARP expression was quantified. (**M**) GNM cells’ cleaved caspase 3 expression was quantified. (**N**) GNM cells’ cleaved caspase PARP expression was quantified. (**O**) Cytochrome c protein expression in mitochondria in SAS cells. (**P**) Cytochrome c cytosol protein expression in SAS cells. (**Q**) SAS cell mitochondria and cytoplasm protein expression of cytochrome c quantitation. (**R**) Cytochrome c protein expression in mitochondria in GNM cells. (**S**) Cytochrome c cytosol protein expression in GNM cells. (**T**) Quantification of mitochondrial and cytosol protein expression of cytochrome c in GNM cells. The mean and standard deviation are used to express the values. Tukey’s multiple range test statistical analysis was used to assess the significance of differences in weeks across different tests. Significant differences between groups were denoted by different letters: a, b, and c, indicate that the results are statistically different (*p* < 0.05).

Figure 1 shows that excessive ALA concentrations induce cell death. The cells were treated with 0, 50, 100, and 200 μM of ALA and 0, 100, 200, and 400 μM of ALA, respectively, and the expression of apoptosis-related proteins was detected to elucidate the potential pathways by which ALA promotes apoptosis in SAS and GNM cells. In addition, apoptosis induction by ALA was verified using flow cytometry when the cells were treated with 0, 50, 100, 200, and 400 μM and 0, 100, 200, 400, and 600 μM of ALA for 24 h, respectively. Figure 4A–D demonstrates that the percentage of apoptotic cells increased with the increasing ALA concentration, with statistically significant differences being seen between the 200 μM and 400 μM of ALA-treated SAS cells (Figure 4C) and the 400 μM and 600 μM of ALA-treated GNM cells (Figure 4D). To further investigate the effects of ALA on cell shape and apoptosis, SAS and GNM cells were treated with 200 μM and 400 μM of ALA for 0, 12, and 24 h, respectively. After 24 h of ALA treatment, both SAS and GNM cells shrank noticeably and showed an increase in the number of floating cells (Figure 1A–D), with an accompanying rise in the number of apoptotic cells (Figure 4C,D). This demonstrates that within 24 h, ALA promotes apoptosis in SAS and GNM cells. Cleaved caspase 8 (Figure 4F,I), cleaved caspase 9 (Figure 4G,J), and cleaved caspase 3 (Figure 4K,M) protein expression was shown to increase with the increasing ALA dose (Figure 4E–N). Cleaved caspase PARP was most strongly induced in SAS and GNM cells by 200 μM and 400 μM of ALA, respectively (Figure 4L,N). According to these findings, ALA has the ability to trigger apoptosis in SAS and GNM cells via endogenous mechanisms. The cells were treated with 0, 50, 100, and 200 μM of ALA and 0, 100, 200, and 400 μM of ALA, respectively, and the expression of mitochondrial and cytoplasmic cytochrome c proteins was detected to assess if ALA regulates the endogenous apoptotic pathway to start apoptosis in SAS and GNM cells. The results indicate (Figure 4O–T) that the amount of cytochrome c in the mitochondria greatly decreased and the amount of cytochrome c in the cytoplasm significantly increased as the dose of ALA was raised. It is believed that ALA promotes cell death by inducing mitochondrial permeability, which in turn causes the release of cytochrome c from the mitochondria into the cytoplasm.

### 3.4. ALA’s Ability to Regulate the Expression of Fas, FasL, Bid, and Apoptosis-Related Proteins in SAS and GNM Cells

**Figure 5:**
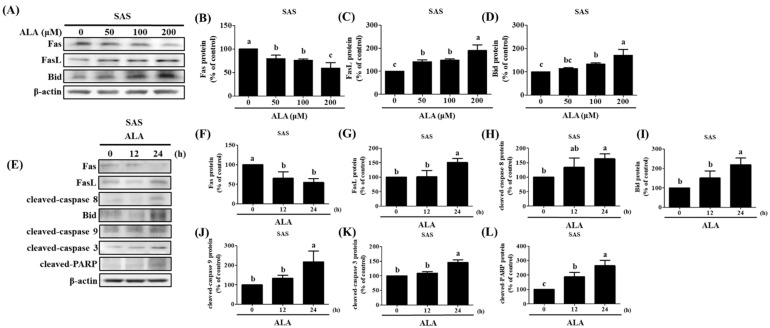
The effect of ALA on Fas, FasL, Bid, and apoptosis-related protein expression in SAS cells at various time points and ALA concentrations. (**A**) Fas, FasL, and Bid expression in SAS cells at various ALA concentrations. (**B**) Quantification of Fas expression in SAS cells at various ALA doses. (**C**) SAS cell FasL expression measurement under various ALA concentrations. (**D**) Bid expression measurement of SAS cells at various ALA concentrations. (**E**) Fas, FasL, Bid, and apoptosis-related protein expression in SAS cells at various time points after ALA treatment. (**F**) Quantification of Fas expression in SAS cells at various time points after ALA treatment. SAS cell (**G**) FasL expression measurement at various time points after ALA treatment. (**H**) Cleaved caspase 8 expression in SAS cells at various time points after ALA treatment. (**I**) Bid expression measurement of SAS cells at various time points after ALA treatment. (**J**) Quantification of cleaved caspase 9 expression in SAS cells at various time periods after ALA treatment. (**K**) SAS cell cleaved caspase 3 expression measurement at various time points after ALA treatment. (**L**) Quantification of cleaved PARP expression in SAS cells at various time periods after ALA treatment. The mean and standard deviation are used to express the values. Tukey’s multiple range test statistical analysis was used to assess the significance of differences in weeks across different tests. Significant differences between groups were denoted by different letters: a, b, and c, indicate that the results are statistically different (*p* < 0.05).

**Figure 6:**
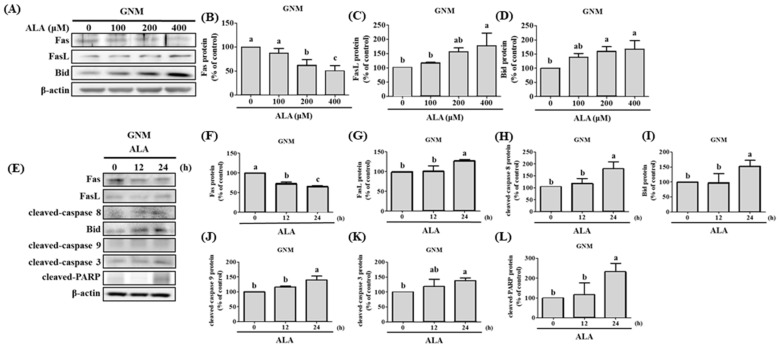
The effect of ALA on Fas, FasL, Bid, and apoptosis-related protein expression in GNM cells at various time points and ALA concentrations. (**A**) Fas, FasL, and Bid expression in GNM cells at various ALA concentrations. (**B**) Quantification of Fas expression in GNM cells at various ALA doses. (**C**) GNM cell FasL expression measurement under various ALA concentrations. (**D**) Bid expression measurement of GNM cells at various ALA concentrations. (**E**) Fas, FasL, Bid, and apoptosis-related protein expression in GNM cells at various time points after ALA treatment. (**F**) Quantification of Fas expression in GNM cells at various time points after ALA treatment. GNM cell (**G**) FasL expression measurement at various time points after ALA treatment. (**H**) Cleaved caspase 8 expression in GNM cells at various time points after ALA treatment. (**I**) Bid expression measurement of GNM cells at various time points after ALA treatment. (**J**) Quantification of cleaved caspase 9 expression in GNM cells at various time periods after ALA treatment. (**K**) GNM cell cleaved caspase 3 expression measurement at various time points after ALA treatment. (**L**) Quantification of cleaved PARP expression in GNM cells at various time periods after ALA treatment. The mean and standard deviation are used to express the values. Tukey’s multiple range test statistical analysis was used to assess the significance of differences in weeks across different tests. Significant differences between groups were denoted by different letters: a, b, and c, indicate that the results are statistically different (*p* < 0.05).

Previous studies have shown that extrinsic apoptosis in oral cancer cells can be induced by upregulating Fas/FasL/Bid expression [36,37]. Fas, FasL, and Bid protein expression was measured after the cells were treated with varying concentrations of ALA for 24 h. The results reveal that both SAS cells (Figure 5A–D) and GNM cells (Figure 6A–D) expressed less Fas protein and more FasL protein when the dose of ALA increased. The expression of the protein Bid was also boosted by ALA. Initiating extrinsic apoptosis in SAS and GNM cells may be possible due to ALA’s potential to activate caspase 8 via the upregulation of Fas/FasL. ALA may also induce intrinsic apoptosis in SAS and GNM cells by elevating Bid protein expression, followed by further Bid activation via caspase 8. Figure 4E–N, Figure 5 and Figure 6 show that ALA can cause apoptosis in SAS and GNM cells via both extrinsic and intrinsic apoptotic mechanisms, respectively. To further investigate the influence of the ALA treatment period on the expression of apoptosis-related proteins, SAS and GNM cells were treated with 200 μM and 400 μM of ALA for 0, 12, and 24 h. Figure 5E–L and Figure 6E–L show that as time went on, ALA inhibited Fas expression and induced both FasL protein expression and the expressions of cleaved caspase 8 (Figure 5H) and Bid (Figure 5I), cleaved caspase 9 (Figure 5J), cleaved caspase 3 (Figure 5K), and PARP (Figure 5L). The 24 h ALA treatment group showed the most dramatic improvements. Within 24 h, ALA induced Fas/FasL-mediated apoptosis in both SAS and GNM cells.

### 3.5. The Effects of Different Treatment Times of ALA on the Expression of Phosphorylated JNK Protein, Nuclear c-jun Protein Accumulation, and Their Association with FasL in SAS and GNM Cells

**Figure 7:**
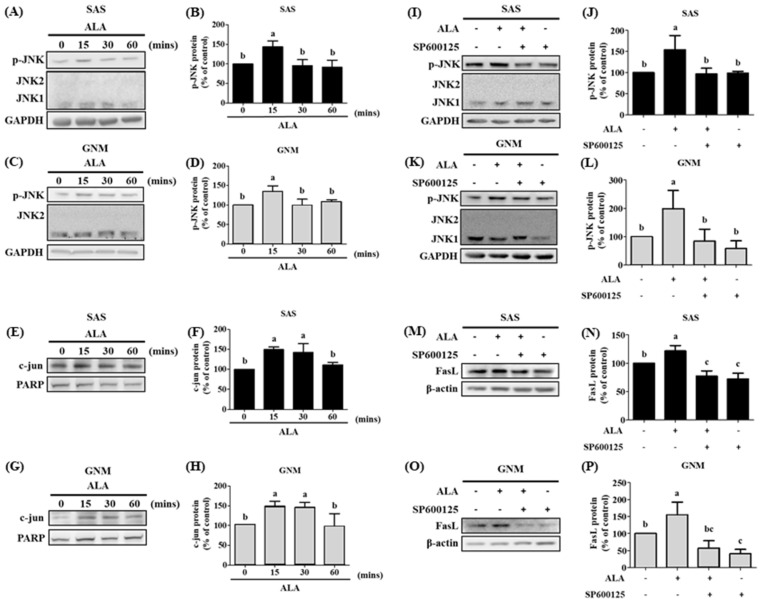
The effect of ALA on p-JNK protein expression and c-jun protein accumulation of nucleus and JNK inhibitor on p-JNK and FasL protein expression in SAS and GNM cells at different time points. SAS and GNM cells were exposed to 200 or 400 μM of ALA for 0, 15, 30, and 60 min. (**A**) p-JNK, JNK1, and JNK2 protein expression in SAS cells at various time periods after ALA treatment. (**B**) Quantification of p-JNK protein expression in SAS cells at various time periods after ALA treatment. (**C**) p-JNK, JNK1, and JNK2 protein expression in GNM cells at various time periods after ALA treatment. (**D**) Quantification of p-JNK protein expression in GNM cells at various time points after ALA treatment. (**E**) c-jun and PARP protein expression in SAS cells at various time periods after ALA treatment. (**F**) Quantification of c-jun protein expression in SAS cells at various time periods after ALA treatment. (**G**) c-jun and PARP expression in GNM cells at various time periods after ALA treatment. (**H**) Quantification of c-jun and protein expression in GNM cells at various time periods after ALA treatment. (**I**) Protein expression of p-JNK, JNK1, and JNK2 in SAS cells after treatment with a JNK inhibitor. (**J**) Quantification of p-JNK protein expression in SAS after treatment with a JNK inhibitor. (**K**) p-JNK, JNK1, and JNK2 protein expression in GNM cells after JNK inhibitor treatment. (**L**) Quantification of p-JNK protein expression in GNM cells after treatment with a JNK inhibitor. (**M**) FasL protein expression in SAS cells after JNK inhibitor treatment. (**N**) FasL protein expression quantification in SAS cells after JNK inhibitor treatment. (**O**) FasL protein expression in GNM cells after JNK inhibitor treatment. (**P**) FasL protein expression quantification in GNM cells after JNK inhibitor treatment. Western blotting was used to determine protein expression. The mean and standard deviation are used to express the values. Tukey’s multiple range test statistical analysis was used to assess the significance of differences in weeks across different tests. Significant differences between groups were denoted by different letters: a, b and c indicate that the results are statistically different (*p* < 0.05).

Previous research has linked c-jun overexpression and FasL upregulation to JNK signaling activity [38]. A significant association was found between JNK and FasL when analyzing samples from clinical head and neck squamous cell carcinoma patients using the cBioPortal gene database, which shed light on the nature of the interaction between JNK and FasL [39]. Additionally, JNK expression in oral cancer was analyzed using GENT2 (Gene Expression database of Normal and Tumor tissues 2). In contrast to normal tissues, tumor tissues were shown to express JNK at much lower levels [40]. SAS and GNM cells were treated with 200 μM and 400 μM of ALA for 0 (control), 15, 30, and 60 min, respectively, and their expression of p-JNK and nuclear c-jun proteins were analyzed using Western blotting (Figure 7A–H). Figure 7A–D shows that both the p-JNK protein expression was enhanced after 15 min of ALA treatment compared with the 0 min ALA treatment group and the c-jun protein accumulation in the nucleus was considerably raised between 15 and 30 min of ALA therapy (Figure 7E–H). These findings provide further support for the hypothesis that FasL protein expression can be upregulated via the activation of the JNK signaling pathway. In this investigation, we used the JNK inhibitor SP600125 to learn more about the connection between JNK and FasL. The data demonstrate that p-JNK expression was higher in the ALA-treated group compared with the control group. Both p-JNK (Figure 7I–L) and FasL expression (Figure 7M–P) were dramatically suppressed via pretreatment with SP600125 in response to ALA. In conclusion, the data presented above demonstrate that ALA stimulates cell apoptosis via the regulation of FasL via JNK activation.

## 4. Discussion

The basic structure of the cell membrane is lipids, with PUFAs, especially n-6 PUFA arachidonic acid (AA), being one of the most essential components [41]. AA can be converted into eicosanoids using phospholipase A2 (PLA2), which are linked to tumor growth, progression, and metastasis [42,43]. Indeed, ALA is classified as an essential fatty acid. However, the conversion of ALA into EPA and DHA is relatively limited, with only approximately 5–10% of ALA being transformed into EPA and about 2–5% into DHA in healthy adults [44]. DHA and EPA have been proven helpful in many studies of anti-cancer properties [45], but ALA has received less attention. Thus, we first want to investigate the tumor-suppressing mechanism of ALA essential fatty acids in the context of oral cancer. Researchers have found that n-3 PUFAs influence immunological responses, inflammation, cell proliferation, apoptosis, metastasis, and angiogenesis in cancer cells by blocking the production of prostaglandins [46]. n-3 PUFAs have been shown to modulate membrane fluidity or modify the structure and composition of lipid rafts, thereby inhibiting the growth of breast cancer cells [47]. DHA ameliorates the Toll-Like Receptor (TLR) 22-triggered inflammation in fish by disrupting lipid raft formation [48]. The supplementation of cells with ALA brought about a significant increase in ALA itself in lipid rafts [49]. These results indicate that ALA may have a potential role in modulating the composition of lipid rafts, subsequently ameliorating TLR-mediated inflammation. Free Fatty Acid receptor 4 (FFA4) (also known as GPR120), recognized as a long-chain fatty acid receptor, is agonized by n-3 PUFAs, including ALA [50]. These FFA4 agonists initiate anti-proliferative and anti-migration effects in various types of cancer [51]. These results indicate the possibility that ALA may enhance FFA4-mediated anti-cancer effects by altering lipid raft function, especially by altering its localization into lipid rafts.

Dietary ALA reduces COX-2 expression and induces apoptosis in hepatoma cells [52]. In ACI-T mice implanted with subcutaneous hepatoma 3924A cells, a diet high in 10% ALA for 28 days significantly suppressed the expression of fatty acid synthase and promoted death in tumor tissues [52]. In BALB/c mice with MCF-7 human breast cancer xenografts and thymectomy, feeding them ALA-rich flaxseed oil (40 g/kg) for 8 weeks inhibited tumor growth and induced apoptosis [53]. A recent study found that ALA inhibits the growth of human breast cancer cells (MCF-7 and MDA-MB-231), as well as cervical cancer cells (SiHa and HeLa), by lowering the generation of nitric oxide (NO) and triggering lipid peroxidation [54]. ALA can also stabilize HIF-1 expression in MCF-7 breast cancer cells and downregulate fatty acid synthase to initiate mitochondrial apoptosis [27]. Although numerous studies have verified ALA’s anticancer properties, its effects on oral cancer are still unknown.

In this study, SAS and GNM cells were used as experimental models to investigate the anticancer effects and mechanisms of ALA. A total of 24 h were spent treating cells with variable concentrations of ALA (0, 25, 50, 100, 200, 400, 600, and 800 μM). It was discovered that 100 and 200 μM of ALA did not induce cytotoxicity in SAS and GNM cells, whereas 200 and 400 μM of ALA significantly reduced cell viability. In the 800 μM ALA treatment group, only about 5% of the cells were viable (Figure 1). Consequently, non-lethal concentrations of ALA (50, 100, and 100, 200 μM) were chosen for further study of their effects on cell migration. As shown in Figure 2, the treatment of SAS and GNM cells with 100 and 200 μM of ALA for 24 h, respectively, inhibited migration and invasion. These results suggest that GNM cells are more malignant than SAS cells because they require larger amounts of ALA to prevent migration and invasion. Upon exposure to ALA at a consistent concentration of 100 μM, SAS cells exhibited a marked decrease in both cell viability and colony formation, while GNM cells did not exhibit a similar inhibitory response (Figure 1). Moreover, the percentage of apoptotic cells significantly increased in SAS cells treated with 200 μM of ALA, while the same effect was observed in GNM cells treated with 400 μM of ALA (Figure 4). These results indicate that the anti-cancer effects of ALA depend on the specific OSCC cell lines.

Human osteosarcoma (MG63, 143B, and U2OS) cell proliferation and invasion were demonstrated to be suppressed with a treatment of 80 μM of ALA in a previous study [55]. The proliferation of breast cancer cells (MCF-7, BT-474, MDA-MB 231, and MDA-MB 468) can be suppressed by ALA at concentrations ranging from 20 to 200 μM by downregulating the expression of proteins such as cyclin-D1, progesterone receptor, and caveolin-1, and by upregulating the expression of estrogen receptor [56,57]. SiHa and HeLa human cervical cancer cell migration can be blocked by ALA (10–80 μM) by the downregulation of VEGF, MMP-2, and MMP-9 expression [31]. Human esophageal cancer cell lines OE19 and OE33 had their proliferation, colony size, adhesion, and migration suppressed by 0.5–5 mM of ALA because of its ability to activate the AMPK signaling pathway and increase the expression of tumor suppressor genes p53, p21, and p27 [58]. Different cell lines have varying ALA tolerances and ALA inhibits cancer cell proliferation and metastasis by regulating diverse signaling pathways. The downregulation of E-cadherin and the upregulation of N-cadherin, fibronectin, and vimentin expression has been linked to Twist1, which is one of the primary transcription factors that induce EMT. Studies show that E-cadherin is downregulated and Twist is overexpressed in OSCC tumor tissues relative to normal tissues [59]. The nuclear expression of Twist is significantly correlated with clinical stage and lymph node metastasis in OSCC cells compared with normal oral mucosal cells [34], suggesting that Twist plays a crucial role in the development and lymph node metastasis of OSCC. The nuclear expression of Twist is significantly correlated with clinical stage and lymph node metastasis in OSCC cells compared with normal oral mucosal cells [34], indicating that Twist plays a crucial role in the development and lymph node metastasis of OSCC. Recent work in our group found that ALA suppresses EMT, migration, and invasion of MDA-MB-231 human breast cancer cells [60]. However, it is unclear if ALA controls EMT in human oral squamous cell carcinoma cells. Figure 3 shows that a dose-dependent inhibition of Twist expression was seen when ALA (100 μM and 200 μM) was applied to SAS and GNM human oral squamous cell carcinoma cells. The expression of Vimentin was downregulated, while that of E-cadherin was upregulated, as a result of ALA therapy. 

Oral cancer cell migration and invasion can be encouraged by elevated MMP-2 and MMP-9 expression and activity [35]. The expression and enzymatic activity of MMP-2 and MMP-9 in SAS and GNM cells are suppressed by ALA, as shown in Figure 5. These results suggest that ALA can reduce the expression and activity of MMP-2 and MMP-9, promote the expression of E-cadherin, and prevent the migration and invasion of SAS and GNM cells. DHA has been proven in studies to reduce MMP-9 production and activity in MCF-7 human breast cancer cells by blocking the DNA-binding activity of NF-B and AP. More research is needed to confirm whether or not ALA can suppress MMP expression in SAS and GNM cells by modulating NF-B and AP-1 [61].

Auto-activation of procaspase-8, caspase-3, and PARP activation are all known to follow FasL interaction with the Fas receptor and begin extrinsic apoptosis [23]. However, the release of cytochrome c and the activation of the intrinsic apoptosis pathway are both triggered by the cleavage of Bid, which occurs upon caspase-8 activation, to generate tBid, which then inserts into the mitochondrial membrane. As a result, Bid has been singled out as an integral connector between extrinsic and intrinsic pathways [62]. Previous studies have shown that the caspase-8/Bid pathway can trigger apoptosis in oral cancer cells [37]. Increased cell proliferation and invasion capabilities, as well as a poor prognosis, have all been linked to a low FasL/Fas ratio in patients with OSCC [63]. ALA was administered at various concentrations to SAS and GNM human oral squamous cell carcinoma cells. High concentrations of ALA substantially decreased Fas expression and increased FasL protein expression, resulting in the activation of caspase-8 and extrinsic apoptosis (Figure 5 and Figure 6). Additionally, ALA treatment further activates Bid via caspase-8, resulting in intrinsic apoptosis. These results are consistent with Lee et al.’s findings [36]. 

FasL is known to be expressed by T cells during immune responses; it can bond to Fas on the membranes of tumor cells and induce apoptosis. There are two forms of FasL: membrane-bound FasL (mFasL) and soluble FasL (sFasL). mFasL is cleaved by MMP to produce the latter [64]. Increased sFasL can compete with mFasL on T cells to bind to Fas receptors on the membranes of cancer cells, thereby inhibiting the signaling that induces cell apoptosis and suppressing the immune system’s attack on malignancies [63]. Juniperus communis extract can promote apoptosis in OSCC cells via the exogenous Fas/FasL pathway [36]. The findings of this study demonstrate that ALA effectively inhibited the expression of MMPs, suggesting that ALA may also inhibit the production of sFasL to reduce competitive binding with membrane-bound mFasL. Through the interaction between mFasL and Fas receptors on cancer cells, ALA may induce apoptosis. A previous study has demonstrated that JNK can upregulate the expression of FasL and induce apoptosis in OSCC cells [38]. NF-kB, Sp1, IFN, c-Myc, FKHRL1, and c-jun are only some of the transcription factors that can induce FasL production [34,64]. In the present study, pretreatment with SP600125 substantially inhibited ALA-induced phosphorylation of JNK and the expression of FasL (Figure 7), suggesting that ALA promotes apoptotic cell death via the induction of the JNK signaling pathway and subsequently upregulates FasL expression.

In a 4-week human investigation, participants consumed 6 g of flaxseed oil cake (containing approximately 5.74 g of ALA) daily; at the conclusion of the study, their plasma ALA concentration significantly increased from 85.13 μM to 249.97 μM [65]. On the basis of these findings, it can be estimated that to increase the plasma ALA concentration by 100, 200, or 400 μM, an additional daily intake of 3.75, 7.5, or 15 g of flaxseed oil (each containing 3.6, 7.2, or 14.4 g of ALA) for 4 weeks would be necessary to achieve the target values, which could improve the prognosis of patients with oral squamous cell carcinoma. In previous human trials, 30 mL of flaxseed oil was administered for 4 weeks, and in another study, flaxseed pastries with a high flaxseed content (containing 59% ALA, approximately 23.6 g) were consumed for 30 days [66]. It has been reported that 50 g of flaxseed consumed daily has no negative effects on the human organism [67]. Multiple studies have found that ALA can prevent the migration and invasion of SAS and GNM oral squamous cell carcinoma cells and downregulate the expression of Twist and EMT-related proteins. Apoptosis in SAS and GNM oral squamous cell carcinoma cells can be induced by ALA via the elevation of FasL expression and the activation of the FasL/caspase signaling pathway. The use of ALA as a targeted therapeutic in clinical settings is feasible. Additional studies can be conducted to discover whether ALA can influence the development of oral squamous cell carcinoma via other signaling pathways, and more in-depth animal tests can be performed, all with the hope that ALA can serve as an auxiliary therapy for OSCC patients.

## 5. Conclusions

**Figure 8:**
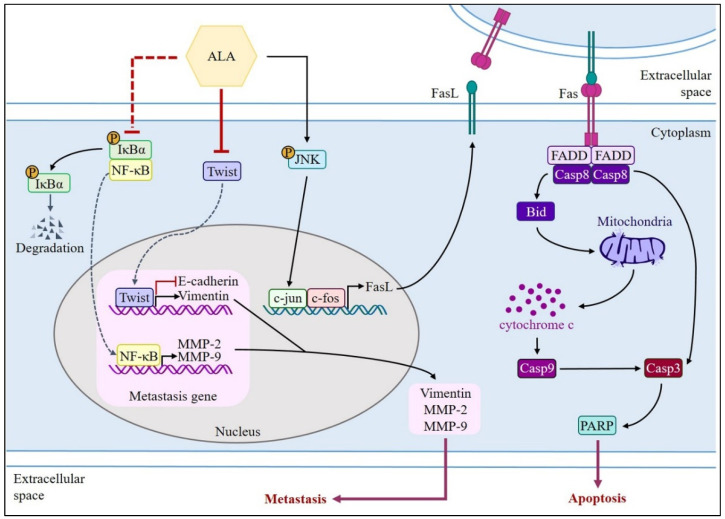
Schematic diagram summarizing the inhibition of ALA on MMP-2 and MMP-9 activity as well as Twist-mediated induction of Vimentin and the suppression of E-cadherin, contributing to cell metastasis. Moreover, ALA can also induce apoptotic cell death via the induction of FasL/caspase 8/caspase 3-extrinsic apoptotic pathway and the Bid/cytochrome c/caspase 9/caspase 3-intrinsic apoptotic pathway in SAS and GNM cells.

The findings of this study are presented schematically in Figure 8. By decreasing the expression and activity of MMP-2 and MMP-9 and Twist and EMT-related proteins, ALA prevents SAS and GNM cells from migrating and invading. ALA can also induce apoptotic cell death by upregulating FasL via the FasL/caspase 8/caspase 3 pathway and the Bid/cytochrome c/caspase 9/caspase 3 pathway in SAS and GNM cells.

## Data Availability

The data presented in this study are available upon request from the corresponding author.
